# Impact of CPAP treatment for a short period in moderate-to-severe OSAS patients: a randomized double-blind clinical trial

**DOI:** 10.1016/j.bjorl.2020.12.011

**Published:** 2021-01-21

**Authors:** Jefferson Luis de Barros Phys, Willian Caetano Rodrigues, Antônio Carlos Marão, Letícia Cláudia de Oliveira Antunes, Sérgio Henrique Kiemle Trindade, Silke Anna Theresa Weber

**Affiliations:** a Universidade Estadual Paulista (UNESP), Faculdade de Medicina, Fundamentos de Cirurgia, Botucatu, SP, Brazil; b Universidade Estadual Paulista (UNESP), Faculdade de Medicina, Departamento de Oftalmologia, Otorrinolaringologia e Cirurgia de Cabeça e Pescoço, Botucatu, SP, Brazil; c Universidade Estadual Paulista (UNESP), Faculdade de Medicina, Botucatu, SP, Brazil; d Universidade Estadual Paulista (UNESP), Faculdade de Medicina, Pediatria, Botucatu, SP, Brazil; e Universidade de São Paulo (USP), Faculdade de Odontologia, Bauru, SP, Brazil; f Universidade Estadual Paulista (UNESP), Faculdade de Medicina, Departamento de Oftalmologia, Otorrinolaringologia e Cirurgia de Cabeça e Pescoço, Divisão de Otorrinolaringologia, Botucatu, SP, Brazil

**Keywords:** Sleep apnea, OSAS, CPAP, Physical activity, Sleep quality, Sleepiness

## Abstract

**Introduction:**

Obesity is the most frequent reversible agravating factor of obstructive sleep apnea syndrome, with physical activity very important for its control. Continuous positive air pressure during sleep is the “gold standard” treatment for obstructive sleep apnea syndrome.

**Objective:**

we aimed to investigate if the use of continuous positive air pressure for a short period (7 days), would improve sleep quality, daytime sleepiness, and the disposition for physical activity.

**Methods:**

Eighty obstructive sleep apnea syndrome patients were randomly assigned as follows: group I – continuous positive air pressure with a steady pressure of 4 cm H_2_O; group II – ideal therapeutic pressure. After filling out the questionnaires related to the studied variables (International physical activity questionnaire long-form, Epworth sleepiness scale, Pittsburgh sleep quality index), patients underwent a baseline pulmonary function test and continuous positive air pressure titration. After continuous positive air pressure therapy for 4≥ hours a night for 7 consecutive days, patients returned and filled out new (International physical activity questionnaire long-form, Epworth sleepiness scale, Pittsburgh sleep quality index) forms. New spirometry was carried out.

**Results:**

39 patients completed the study. The mean age was 52 ± 11 years old and 28 patients (71.79%) were obese. Both groups were similar for all variables studied at baseline. After Continuous positive air pressure use, patients of group II presented more significant improvements (*p* <  0.05) for sleep quality and diurnal sleepiness. Time spent with physical activities did not change. Spirometric data were at normal range at baseline. Solely the variable FEF 25%–75% was significantly enhanced (*p* <  0.05) in group II.

**Conclusion:**

Continuous positive air pressure therapy for 1 week, with ideal pressure, improves daytime sleepiness and sleep quality, enhances pulmonary function, but does not change the mean time spent with physical activities.

## Introduction

Obstructive sleep apnea (OSA) is characterized by recurrent events of partial or complete pharyngeal collapse during sleep. These intermittent events can cause airflow interruption and the inspiratory efforts to overcome airway occlusion lead to awakening, sleep fragmentation, and oxyhemoglobin desaturation.^1^ The term “obstructive sleep apnea syndrome” (OSAS) is preferentially used when OSA events are associated with excessive daytime sleepiness^2^ and/or other common signs and symptoms such as chronic high snoring, nocturnal enuresis, fatigue, non-refreshing sleep, morning headache, decreased concentration, irritability, personality changes and impaired quality of life.^3^, ^4^

The severity of OSAS is influenced by the anatomic susceptibility of the upper airway to collapse along with pharyngeal dilator muscle responsiveness and other factors, such as obesity.^5^ The apnea-hypopnea index (AHI) is the most commonly reported polysomnographic parameter used to objectively diagnose and assess OSAS severity: obstructive AHI 5–15 episodes. h^–1^ (mild); AHI > 15–30 episodes.h^–1^ (moderate); AHI > 30 episodes.h^–1^ (severe).^2^ In children, the indexes are lower: 1–5 (mild); > 5–10 (moderate); 30 or more (severe).^6^

In the early 2000s, a North American study showed that 2% to 4% of middle-aged individuals had OSAS.^7^ However, there was a substantial increase in prevalence rates over the last years, and according to current epidemiological data, the prevalence of moderate to severe sleep-disordered breathing (AHI ≥ 15) is 10% among 30–49 year-old men, 17% among 50–70 year-old men, 3% among 30–49 year-old women and 9% among 50–70-year old women.^8^ In addition to the high prevalence, OSAS is an independent risk factor for poor health and increased cardiovascular morbidity and mortality, which makes this clinical entity into a growing public health problem. There is strong evidence that OSAS is associated with a higher incidence of stroke, systemic hypertension (HTN), heart failure (HF), atrial fibrillation (AF) and coronary heart disease (CHD).^4^, ^9^

Application of continuous positive airway pressure (CPAP) during sleep is considered the “gold standard” treatment for OSAS and has been shown to improve daytime sleepiness, quality of life, and depressive symptoms, as well as reduce risk for cardiovascular disease and mortality.^10^, ^11^, ^12^ Although CPAP therapy is very efficacious in the treatment of OSAS, by itself it has limited metabolic effects, and poor adherence further compromises its effectiveness, as evidenced by recent randomized controlled trials.^12^, ^13^ Lifestyle modifications involving physical activity, diet alterations and weight loss may attenuate or prevent metabolic disease and are encouraged as adjunct therapy to the use of CPAP.^5^, ^11^, ^12^ However, it is unclear whether treatment with CPAP results in behavioral modifications.

By developing this prospective study, we sought to evaluate whether the use of CPAP for a short period (7 days) would be effective in reducing diurnal sleepiness, increasing sleep quality and ameliorate disposition to physical activity in adult patients diagnosed with moderate-to-severe OSAS (AHI ≥ 15). In addition, we also tested whether CPAP treatment would lead to an improvement in the lung function of these patients, specifically in the spirometric fraction FEF 25%–75%.

## Methods

This study was a double-blind randomized clinical trial and followed all ethical principles and recommendations of the Helsinski Declaration of 1964. The protocol was approved by the Research Ethics Committee of Botucatu Medical School, São Paulo State University “Júlio de Mesquita Filho”, Brazil (approval nº 041-2013), and all recruited subjects provided written informed consent before entry.

The population involved consisted of adult patients, without distinction of sex, consecutively referred to the Laboratory of Sleep-disordered Breathing (LSDB) at the above-mentioned institution. Only those with a clinical diagnosis of OSAS, as defined by the American Academy of Sleep Medicine (AASM) criteria,^2^ and AHI ≥ 15 events.h^−1^ by polysomnography (PSG) were included in the sample. The following exclusion criteria were applied: (1) neuropathies; (2) pregnancy; (3) smokers; (4) chronic obstructive pulmonary disease (COPD); (5) previous CPAP use; (6) unstable angina; (7) myocardial infarction; (8) previous cardiac surgery.

A total of 80 patients who met the criteria were invited to enroll in the study and were randomly assigned to the following two groups: group I (n = 40): CPAP equipment calibrated to release a steady pressure of 4 cm H_2_O; group II (n = 40): CPAP equipment previously calibrated to release therapeutic pressure (cm H_2_O), sufficient to maintain AHI < 5, which was determined during titration PSG.

In the first interview, complete demographic data and written informed consent were obtained. Patients filled in three subjective questionnaires (described below) and underwent a baseline spirometry test to assess lung function prior to CPAP use. In the second appointment, an initial CPAP titration was done at the LSDB, in order to establish the ideal therapeutic pressure to avoid OSA events. Unfortunately, 15 patients from group I and 19 from group II did not attend at least one of these appointments for baseline data collection. Therefore, they were excluded from the study.

The information on physical activity patterns, before and after CPAP use, were gathered using the international physical activity questionnaire long-form (IPAQ-L), proposed by World Health Organization (WHO) in 1998, as an instrument for public health surveys.^14^ This questionnaire is divided into five sections in which all physical activities are reported, including those related to work environment, means of transport, domestic tasks, sports and leisure practices. Lastly, time spent sitting is also evaluated.^15^ The totals for each domain were summed and thus, the total level of physical activity was calculated in minutes per week. IPAQ-L also enables the evaluation of physical activity energy expenditure and provides the amount of activity in metabolic equivalents or ratio of work metabolic rate to a standard metabolic rate (MET). One MET is roughly equivalent to the energy cost of sitting quietly or 0.0175 calories per minute per kilogram of body weight (kcal/min/kg).^14^ For measurement of energy expenditure with the activities in each session, the following formula was used: kcal = activity MET × body weight (kg)/60 × time of activity (min).

Diurnal hypersomnolence, an important symptom of OSAS, was measured using the Epworth sleepiness scale (ESS), which is a self-administered questionnaire designed based on observations related to the nature and occurrence of daytime sleepiness. It assesses the probability of falling asleep in eight different situations involving daily activities, some of them known to be highly soporific. ESS averages the responses to derive a total score; higher scores represent a higher level of subjective sleepiness.^16^

Application of the Pittsburgh sleep quality index (PSQI) allowed us to establish a sleep disorder severity index and was measured before and after CPAP use. This questionnaire was structured for the evaluation of sleep quality in the previous month and consists of 19 questions grouped into seven components, each scored on a scale of 0 to 3.^17^ These components are, respectively: subjective quality of sleep; sleep latency; sleep duration; habitual efficiency; sleep disorder; using medications for sleep; and diurnal dysfunction. The scores of the seven components are added together to give an overall score ranging from 0 to 21. A global PSQI score > 5 points indicate major difficulties in at least 2 components, or moderate difficulties in at least 3 parameters.^18^

Pulmonary function testing was performed on the first and in the eighth day of the study using a computerized spirometer (Ferraris KOKO, Louisville, CO, USA®). The best forced vital capacity (FVC) maneuver from at least 3 attempts was chosen based on American Thoracic Society criteria, i.e., the highest sum of FEV1 and FVC.^19^ The following spirometry variables were measured: forced vital capacity (FVC); forced expiratory volume in 1 s (FEV1); Tiffeneau-Pinelli index or FEV1/FVC ratio; average forced expiratory flow between 25% and 75% of the FVC curve (FEF 25%–75%); and peak expiratory flow (PEF). To perform the spirometric maneuvers, subjects kept themselves seated, with erect spine, feet resting on the floor and a nasal clip correctly positioned.

CPAP equipment was handed to the patients with concealed displays so that both, patient, and researcher did not have access to the calibration data previously programmed by the study supervisor. Instructions for use were presented in writing and patients were advised to use CPAP for at least four hours a night for seven consecutive days. On the eighth day, patients returned to LDSB and filled out new IPAQ-L, ESS and PSQI forms. Data stored on the memory card of each device allowed the evaluation of correct CPAP use and patients who did not properly adhere to the pre-established study protocol (CPAP use < 4 h per night or who did not use for 7 consecutive days) were also excluded.

STATA® software version 15 (StataCorp LLC, Texas, USA) was used for data analysis. Continuous variables were expressed as mean ± standard deviation (SD). At baseline, Student's *t*-test was used to compare the variables gender and age between the two groups while the non-parametric Kruskal-Wallis test was used to search for significant differences for BMI and AHI.

Means of groups were compared by two-way analysis of variance (ANOVA) for repeated measurements followed by the post hoc Tukey test. A Student's *t*-Test was used for comparisons of two means when appropriate. A *p*-value < 0.05 was considered significant for all statistical analyses.

## Results

**Figure 1:**
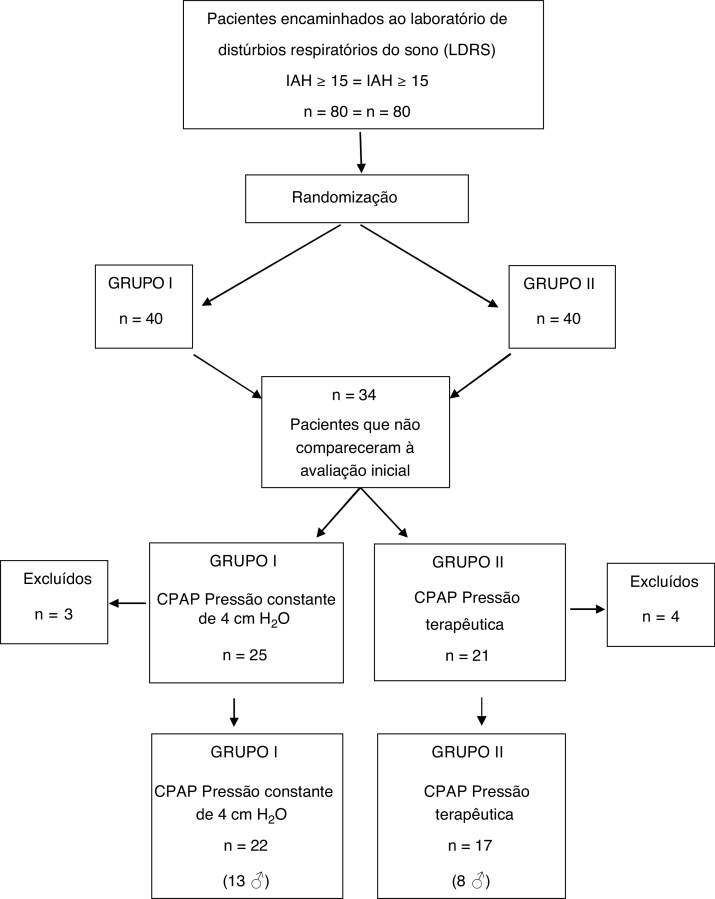
Flowchart of patients’ inclusion and distribution in the respective groups.

Among the 80 patients invited to the study, 34 were excluded still in the early stage, because they did not attend the initial spirometry session and/or CPAP titration. Seven other subjects did not adhere to CPAP use protocol and their data were not part of the analysis. Therefore, the final sample consisted of 39 patients, distributed as follows: 17 patients (8 men) in group I and 22 (13 men) in group II (Fig. 1).

Demographics of all participants, including gender, age, and body mass index (BMI), as well as the respective apnea and hypopnea indexes (AHI) are listed in Table 1. There was a predominance of male patients, over 40 years old and obese (BMI ≥ 30 kg.m^−2^). In this sample, there was a higher prevalence of OSAS in its moderate-to-severe form. Comparing these variables between the two groups, there was no statistically significant difference.

**Table 1:** Comparison of demographics, gender, age, BMI and OSAS severity between the two groups.

	Sample	Group I – steady pressure of 4 cm H_2_O (n = 22)	Group II -therapeutic pressure (n = 17)	*p*^a^
Gender	21♂ e 18♀	13♂ e 9♀	8♂ e 9♀	*p* < 0.05
Age (years)	52 ± 11	54 ± 10	50 ± 11	*p* < 0.05
BMI (kg. m^−2^)	32 ± 4	31 ± 3	32 ± 5	*p* < 0.05
AHI (e. h^−1^)	28 ± 13	25 ± 13	32 ± 14	*p* < 0.05

n, number of patients; BMI, Body Mass Index; AHI, Apnea and Hypopnea Index.

a Student's *t*-test for comparison of gender and age between groups. BMI and AHI were compared using Kruskal-Wallis test.

Data obtained at the beginning of the study, using the IPAQ-L, showed that most patients of our sample, in general, did not perform regular physical activity and spent most of the time sitting. It was found that men spent more time on physical activities related to work while women spent more time on domestic activities. In group II, after the use of CPAP with therapeutic pressure for 7 days, patients reported improvement in their willingness to engage in sports-leisure activities (section 4) with family. However, no effective improvement was observed in the time spent with physical activity. Energy expenditure remained similar to the period prior to the use of CPAP (Table 2). There was no significant relationship between OSAS severity and physical activity patterns.

**Table 2:** Energy expenditure distribution according to the International physical activity questionnaire long-form, before and after CPAP use. Comparison between the two groups.

	Group I – Pressure of 4 cm H_2_O (n = 22)				Group II –Therapeutic pressure (n = 17)			
IPAQ-L	Baseline	kcal	After CPAP	kcal	Baseline	kcal	After CPAP	Kcal
Section 1 work	0.07	588	0.07	556	0.02	180	0.02	180
Section 2 transport	0.07	159	0.07	158	0.06	146	0.06	144
Section 3 domestic	0.08	637	0.07	619	0.10	872	0.08	588
Section 4 leisure	0.03	461	0.03	473	0.04	467	0.04	605
Section 5 sitting	0.49	1,707	0.48	1,669	0.41	1,539	0.39	1,571

Time spent with physical activity expressed in minutes per week. Rate of weekly energy expenditure based on the metabolic equivalent (kcal) = activity MET × body weight (kg)/60 × time of activity (min).

Excessive daytime sleepiness (ESS > 8) was observed in 89.74% of the studied population at baseline: 21 patients in group I (95.4%) and 14 patients in group II (82.3%). After the use of CPAP for one week, only 9.5% of the patients in group I reported improvement in relation to daytime sleepiness, whereas in group II, the increase was statistically significant (*p* <  0.05); 78.6% of the patients did not report the symptom any more. There was a strong correlation between daytime hypersomnolence and low physical activity index (*p* <  0.05). The higher the score found for ESS, the shorter the time spent practicing sports and physical activity during leisure periods.

Regarding the sleep disorder severity, all patients in the sample (100%) had a global PSQI score > 5 points at baseline, which indicates poor sleep quality. After one week of CPAP treatment, 63.6% of the patients in group I presented an improvement in the PSQI index. In group II, the PSQI index was reduced in 94.1% of the patients, a statistically more significant improvement (*p* <  0.05) (Table 3).

**Table 3:** Evaluation of daytime Sleepiness (ESS) and Sleep Quality (PSQI), before and after the use of CPAP for one week. Group I – steady pressure of 4 cm H_2_0. Group II – titrated ideal pressure.

	Group I – Pressure of 4 cm H_2_O (n = 22)			Group II – Therapeutic pressure (n = 17)		
	Baseline	After CPAP	%	Baseline	After CPAP	%
ESS > 8	21	19	9.5	14	3	78.6^a^
PSQI > 5	22	6	63.6^a^	17	1	94.1^a^

ESS, Excessive daytime Sleepiness; PSQI, Pittsburgh Sleep Quality Index; n, total number of patients in the sample; %, Percentage of patients with improvement.

Comparison between groups using ANOVA test^a^ (*p* <  0.05).

Spirometry tests showed all parameters within normal range at baseline, i.e., no patient had obstructive or restrictive respiratory disorders. Solely the variable FEF 25%–75% has undergone statistically significant alterations (*p* <  0.05) after the use of CPAP under titrated therapeutic pressure in the group II (Table 4).

**Table 4:** Mean values and the standard deviations of the main lung function test variables at baseline and after one-week CPAP use for the patients of both groups.

Spirometric variables	Group I – Pressure of 4 cm H_2_O (n = 22)		Group II – Therapeutic pressure (n = 17)	
	Baseline	After CPAP	Baseline	After CPAP
FVC	3.64 ± 1.19	3.66 ± 1.09	3.54 ± 0.83	3.67 ± 0.84
FEV_1_	2.99 ± 1.01	2.97 ± 1.00	2.81 ± 0.69	2.95 ± 0.71
FEV_1_/ FVC	0.81 ± 0.05	0.81 ± 0.05	0.80 ± 0.08	0.80 ± 0.05
FEF 25%–75%	3.04 ± 1.27	3.07 ± 1.40	2.91 ± 1.02^a^	3.13 ± 1.03^a^
PEF	8.01 ± 2.82	7.46 ± 2.58	7.37 ± 1.73	7.37 ± 1.73

FVC, Forced Vital Capacity; FEV_1_, Forced Expiratory Volume in 1 s; FEV1/FVC ratio, Tiffeneau-Pinelli index; FEF 25–75%, Average forced expiratory flow between 25% and 75% of the FVC curve; PEF, Peak Expiratory Flow.

a Comparison by means of Student’s *t*-test.

## Discussion

Demographic data from our sample showed most patients were male, obese and over 40 years old, besides manifesting severe OSAS. Concerning the OSAS severity, we must emphasize that Botucatu Medical School Hospital is a public institution that provides free care to patients in a region whose population is estimated at about 500 thousand inhabitants. In recent years, it has become one of the major referring centers for diagnosis and treatment of sleep disorders. As a result, the demand for care at the Sleep Outpatient Clinic far outweighs its capacity of attendance. This implies the need to prioritize the treatment of more critical patients and may justify higher prevalence of severe OSAS in our study.

The prevalence of OSAS varies considerably by age group, with one peak occurring in 2–6 aged children, related to adenotonsillar hypertrophy or craniofacial abnormalities, after which there is a declining prevalence until early adulthood,^6^ when the rates of OSAS progressively increase into older age where the prevalence is at least 20%.^8^ There is a positive correlation between age, obesity and neck circumference; that is, with aging, increased BMI and neck circumferential size, which are risk factors for OSAS.^13^, ^20^ The caliber of the upper airways decreases with advancing age in both men and women, which can be explained by the abovementioned factors and by the reduction of pharynx dilator musculature activity.^20^, ^21^, ^22^ It has also been shown that OSAS manifested in elderly people brings milder consequences than those in young patients, suggesting that the diagnostic criteria should be adjusted for age.^8^, ^21^, ^22^

Several morphological alterations may compromise the patency of the upper airway, including craniofacial deformities, enlarged tonsils, upper airway edema and decreased lung volume.^23^ However, due to direct mechanical effects on the respiratory system, the major risk factor for OSAS is obesity. Fat deposits within the upper airway reduces its diameter and excessive accumulation of abdominal fat hinders the expansion of the thoracic cavity in the vertical aspect, which decreases lung volume capacity.^5^, ^11^, ^23^ Roughly 20% to 40% of OSAS patients are not obese. In these individuals, nonanatomic factors, such as upper airway dilator muscle dysfunction, heightened chemosensitivity, and low arousal threshold are the main causes and define various phenotypes of OSAS.^23^

Not surprisingly, our study sample showed a smaller number of women. Bixler and colleagues have produced evidence that men are more likely to develop OSAS. Using clinical and polysomnographic criteria, the proportion found was 1.2% of women to 3.9% of men.^22^ This may be explained by increased genioglossus muscle tone in women than in men during sleep, suggesting a defense mechanism for maintenance of upper airways permeability.^20^ In fact, when lying in the supine position, males have a greater reduction in the upper airways’ caliber than females. Another situation that corroborates this hypothesis is that increased neck circumference by fat accumulation reduces the transverse diameter of the upper airways more intensely in men than in women.^20^, ^22^ Moreover, premenopausal women have higher genioglossal muscle activity when compared to postmenopausal women and men of the same age. It is then believed that progesterone may play a protective role in apnea prior to menopause.^20^

Poor sleep quality is a common complaint in OSAS patients. In our study, all patients in the sample (100%) had a global PSQI score > 5 at baseline, indicating major difficulties related to latency, duration, or sleep efficiency. Deterioration of sleep quality presents a significant but weak association, with the deterioration in the perception of quality of life.^24^ The nocturnal sleep restriction, identified in our sample through PSQI nocturnal sleep duration, may result in a sensation of physical and mental fatigue during the day, predisposing to excessive daytime sleepiness. Our results demonstrate that CPAP use of ≥ 4 h per night, for 7 days, among patients with moderate-to-severe OSAS is able to ameliorate significantly the scores in almost all cases of group II (therapeutic pressure). These results are in accordance with previous studies.^24^, ^25^ Even in group I, in which sham CPAP was used, there was a significant improvement in the PSQI score, something that we attributed to the placebo effect. Although snoring and other symptoms are still present, initiating a treatment may induce a subjective perception of improvement in sleep quality.^25^

Excessive daytime sleepiness is an important clinical feature of OSAS and one of the main reasons patient seek medical attention.^3^, ^6^ Similarly to other studies,^16^, ^26^, ^27^ in our sample, daytime sleepiness with high ESS was present in 35 of the 39 OSAS patients. CPAP treatment with therapeutic pressure (group II) led to a significant improvement in subjective daytime sleepiness, despite a short period of use; this was not reproduced by the use of CPAP with non-therapeutic pressure (group I). The maintenance of positive airway pressure prevents pharyngeal collapse and consequent episodes of apnea, inhibiting sleep fragmentation.^9^, ^26^, ^27^

Poor sleep quality and diurnal hypersomnolence may negatively affect some social aspects such as physical activity practice as leisure or for sports.^10^ Indeed, our sample data confirmed this aspect. As mentioned above, obesity is a major cause of OSAS worsening and changes in weight are a reflection of net caloric balance: the difference between caloric expenditures and consumption.^5^ It was suggested that CPAP treatment, by alleviating fatigue and sleepiness, would lead to increased physical activity, energy consumption and, consequently, would promote weight loss.^28^, ^29^ However, some studies have shown that CPAP use is associated with weight gain over periods ranging from 3 to 6 months.^30^, ^31^ And a recently- published study provided strong evidence that using CPAP for a single week leads to weight gain due to the reversal of nocturia and the consequent accumulation of fluid.^32^ Anyway, we speculated that therapeutic CPAP for one week could improve the patterns of physical activity and daily energy consumption of OSAS patients.

For evaluation of physical activity patterns, we used the IPQA-L questionnaire, which validity and reproducibility has already been tested in several countries, including Brazil.^15^ It has been shown to be effective in predicting total energy expenditure in epidemiologic studies.^14^ In the male population, individuals spend most of their time sitting (IPAQ-L – section 5), evidencing a marked sedentary lifestyle in the studied population. Women spend less sitting time and exert, on average, more energy with household chores than men. This is in keeping with Brazilian culture, where historically, mainly women have usually carried out housework. In our sample, the short time of CPAP therapy improved the disposition but not the time spent with physical activity. Lack of commitment to perform physical activities may be related to the short observation period (one week) and to other cultural and social factors not addressed by our study. We believe that more direct interventions by physiotherapists and physical educators would also be important to effectively induce positive changes in life habits.

Regarding the pulmonary function test, all measured variables remained unchanged before and after the use of CPAP in both groups, except for the component FEF 25%–75%, which enhanced in a statistically significant way in the Group II. It is interesting that in this study, we observed a substantial improvement in this spirometric component even after short-term CPAP therapy. This variable depends on the elastic retraction force of the lungs and airway permeability. It is related to involuntary expiration phase without interference of the expiratory muscles and, therefore, is independent of patient collaboration.^19^ For us, it is reasonable to assume that CPAP therapy at ideal therapeutic pressure, even in a short period, has assisted in increasing the involuntary tone of pharyngeal musculature and altered the sensitivity of muscle activation reflex at the onset of respiration as proposed by other researchers.^33^ However, we recognize that further prospective studies with larger samples should be performed to reinforce this hypothesis. In order to evaluate changes in the perfusion/ventilation ratio, a biochemical analysis of blood gases would be necessary, something that we did not include in this study.

There are many limitations in our study. Firstly, there was no power analysis done in order to calculate the number of subjects that need to be recruited in order to accept or refute the authors' hypothesis. Moreover, a significant limitation of CPAP treatment is patients’ adherence, which may be influenced by morphological characteristics, treatment titration procedures, side effects, in addition to psychological and social factors. Approaches that could improve or control these aspects would increase the level of adherence to this treatment modality. Unfortunately, only 39 subjects out of 80 completed the protocol. Despite the randomization process, this may affect the results and introduce bias into the study. Furthermore, we did not perform the intention-to- treat analysis and the non-adherent subjects were not included in the sample.

## Conclusion

In despite of the small sample and the limitations of our study, we can state that CPAP therapy for one single week, with ideal pressure, and at least 4 h per night, apparently improves daytime sleepiness, sleep quality and disposition for physical activity, mostly for sports. Moreover, CPAP treatment for this short period may enhance pulmonary function.

## Conflicts of interest

The authors declare no conflicts of interest.

## Data availability statement

The data that support the findings of this study are available on request from the corresponding author. The data are not publicly available due to privacy or ethical restrictions.
